# Targeting Tumor Microenvironment for Cancer Therapy

**DOI:** 10.3390/ijms20040840

**Published:** 2019-02-15

**Authors:** Catarina Roma-Rodrigues, Rita Mendes, Pedro V. Baptista, Alexandra R. Fernandes

**Affiliations:** UCIBIO, Departamento de Ciências da Vida, Faculdade de Ciências e Tecnologia, Universidade NOVA de Lisboa. Campus de Caparica, 2829-516 Caparica, Portugal; catromar@fct.unl.pt (C.R.-R.); ars.mendes@campus.fct.unl.pt (R.M.)

**Keywords:** Tumor microenvironment, tumor development, cancer therapy, models for tumor microenvironment study, nanomedicines

## Abstract

Cancer development is highly associated to the physiological state of the tumor microenvironment (TME). Despite the existing heterogeneity of tumors from the same or from different anatomical locations, common features can be found in the TME maturation of epithelial-derived tumors. Genetic alterations in tumor cells result in hyperplasia, uncontrolled growth, resistance to apoptosis, and metabolic shift towards anaerobic glycolysis (Warburg effect). These events create hypoxia, oxidative stress and acidosis within the TME triggering an adjustment of the extracellular matrix (ECM), a response from neighbor stromal cells (e.g., fibroblasts) and immune cells (lymphocytes and macrophages), inducing angiogenesis and, ultimately, resulting in metastasis. Exosomes secreted by TME cells are central players in all these events. The TME profile is preponderant on prognosis and impacts efficacy of anti-cancer therapies. Hence, a big effort has been made to develop new therapeutic strategies towards a more efficient targeting of TME. These efforts focus on: (i) therapeutic strategies targeting TME components, extending from conventional therapeutics, to combined therapies and nanomedicines; and (ii) the development of models that accurately resemble the TME for bench investigations, including tumor-tissue explants, “tumor on a chip” or multicellular tumor-spheroids.

## 1. Introduction

**Figure 1:**
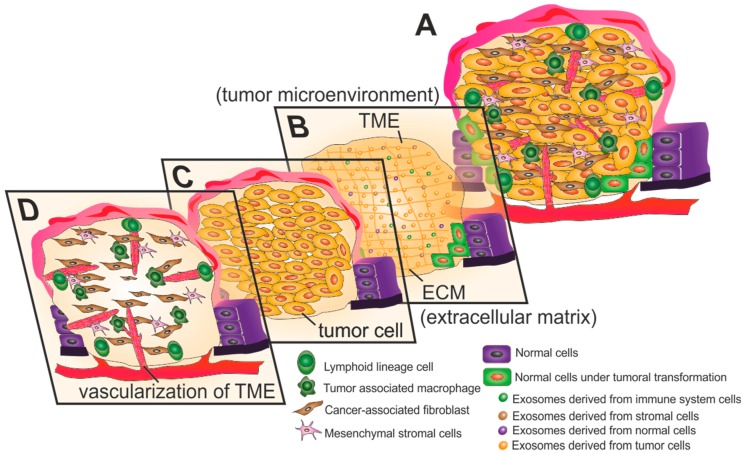
Anatomy of the tumor microenvironment (TME). (**A**) The TME of a late stage solid tumor is highly heterogeneous and complex. (**B**) Exosomes play important roles in paracrine and autocrine communication between TME cells, being preponderant in the modulation and development of the tumor. Exosomes are also involved in the transformation of normal cells adjacent to the TME into tumor cells. The extracellular matrix (ECM) in the TME is frequently dense and stiff, resulting in desmoplasia. (**C**) The rapid growth of tumor cells results in hypoxic regions and lack of nutrients within the tumor, causing the Warburg effect. This metabolic shift into anaerobic glycolytic pathway results in acidification of the TME. (**D**) The rapid growth of tumor cells induces angiogenesis and consequent formation of chaotic branching structures. Stromal cells (including Cancer-associated fibroblasts and mesenchymal stromal cells) and cells from the immune system (both lymphoid and myeloid lineage cells) are important players in tumor development and prognosis.

The development of effective anti-cancer therapies has been challenged by the overall complexity of tumors [1,2,3]. The tumor heterogeneity is exacerbated during the progression of the cancer along with the maturation of the cellular and noncellular components of the tumor niche—the tumor microenvironment (TME) [4,5]. The TME consists of extracellular matrix (ECM), stromal cells (such as fibroblasts, mesenchymal stromal cells, pericytes, occasionally adipocytes, blood and lymphatic vascular networks) and immune cells (including T and B lymphocytes, natural killer cells and Tumor-associated macrophages) [6]. The TME has progressively been shown to dictate aberrant tissue function and to play a critical role in the subsequent evolution of malignancies. Epithelial tumors display common features that allow for the setting of hallmarks that define cancer progression [7,8]. Tumor initiation is based on a complex series of biological events occurring on a normal cell that will result in hyperplasia, uncontrolled growth and resistance to cell death [8,9]. As tumor cells continue proliferation, the tumor increases in size with an associated remodeling of the TME. This is induced by hypoxia, oxidative stress and acidosis, due to an alteration of tumor cells metabolism, resulting in dysplasia, which is the appearance of a heterogeneous population of tumoral cells with different genetic and phenotypic traits [10,11]. These events are orchestrated by autocrine and paracrine communications with stromal cell and immune system adjacent to the tumor, coupled to an increased interstitial fluid pressure [8,11]. Once again, autocrine and paracrine communications between TME cells induce TME maturation and tumor progression, resulting in increased stiffness of the extracellular matrix, formation of blood and lymph vessels, possible appearance of necrotic regions and metastasis [8]. Figure 1 is a schematic representation of the major events of tumor progression. 

Tumor development is highly dependent on the specific TME, which is preponderant on prognosis and impacts chemotherapy efficiency [12]. Understanding how the composition of the TME changes during tumor development may allow for the development of therapeutic strategies able to tackle the tumor at a specific evolutionary stage. The study of TME during tumor development reveals prognostic biomarkers that may be used for imaging or for liquid biopsy analysis, both important to select the most suitable therapy (reviewed in [13,14,15]). This review summarizes the current knowledge on the major players/events involved in TME maturation of the primary tumor that can induce or disrupt cancer progression. Additionally, we discuss therapeutic strategies targeting these events, from standard current therapeutics to nanomedicine-based approaches and future methods to monitor the TME in vitro.

## 2. Targeting the Tumor Microenvironment

Chemotherapy is the leading cancer therapy worldwide, often combined with surgery, or surgery and radiotherapy, depending on tumor stage [16]. Since the discovery of several important mutations that contribute to carcinogenesis (e.g., epidermal growth factor receptor (EGFR), p53, and c-Myc), these have been extensively used as targets for the development of more selective drugs to tackle cancer [17]. Despite the effectiveness of these drugs, multidrug resistance (MDR) has been increasing, which often results in tumor relapse and low quality of life for patients [17]. Cancer research has been focused on tumor cells even though ever more often the effect of the TME has been shown to play a key role in tumor progression and MDR [17]. 

At a late stage solid tumor, the tumor microenvironment is highly complex and heterogeneous. Initially, the genomic profiles of tumor cells are preponderant for the modulation of the tumor vicinity [7,8,9]. The rapid expansion of the tumor cells triggers several events, such as hypoxia that results in a metabolic reprogramming of tumor cell, and an adjustment of TME to the new context [11,18,19,20,21]. The interplay between cancer cells and neighboring cells, including stromal and immune system cells (which frequently appear due to inflammation at the tumor location) results in further alterations of the TME cellular components, restructuration of the extracellular matrix and formation of chaotic vascularization systems, which may lead to metastasis [14]. During tumor growth, cancer cells and TME constituents are continually adapting to the environment conditions, influencing the overall tumor growth. 

**Figure 2:**
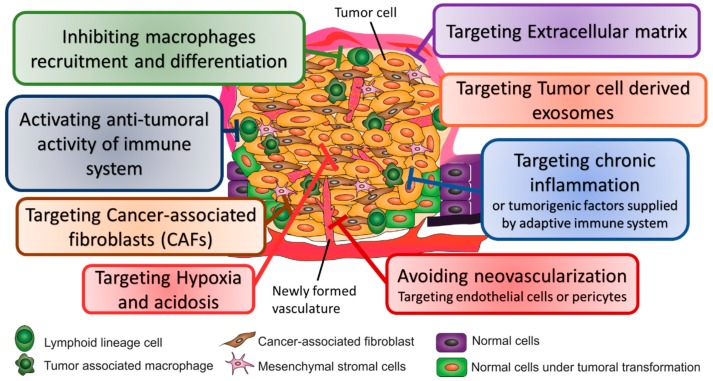
Strategies used to target tumor microenvironment for cancer therapy.

Understanding the major events occurring in the TME that support primary tumor growth and how these events impact the modulation of the environment is of utmost relevance to assist the definition of efficient therapy strategies [22]. Figure 2 highlights current strategies used to target TME components.

### 2.1. Targeting the Extracellular Matrix

The composition of the ECM is of great relevance to the prognosis of a given tumor [23]. The ECM is a three-dimensional structure of collagen, elastin, fibronectin, hyaluronic acid, proteoglycans and glycoproteins that support tissues by encapsulating cells and providing hydration and pH homeostasis [14,24,25]. Moreover, the ECM acts as a reservoir of growth factors [25]. Under normal conditions, the ECM is divided into the interstitial matrix, which involves the stromal cells forming the connective tissue, and the basal membrane, a specialized layer that in epithelial tissue divides epithelial and endothelial cells from the underlying stroma [14,24]. Importantly, the composition, biomechanics and anisotropy of the ECM are tissue-dependent and are tailored for the specific function of an organ [24].

The heterogeneity of tumor cells, the lack of tissue oxygenation or increased inflammation in the TME induce modifications of the ECM protein components, resulting in increased ECM density and stiffness mainly from an increased collagen deposition, the so-called desmoplasia [14,23]. As the tumor progresses, the ECM becomes more disorganized due local modulations of the ECM within the TME [24,26]. The ECM composition plays important roles in tumor progression, by providing tumor cells with sustaining proliferative signals, evading growth suppressors, resisting cell death, enabling replicative immortality, inducing angiogenesis and promoting invasion and metastasis (reviewed in [24]). The degree of desmoplasia is related to disease progression [14,24]. Angiotensin II receptor agonists commonly used for treatment of high pressure, including Food and Drug Administration (FDA) approved Losartan, Candersartan, Olmesartan or Valssartan, were shown to be effective in reducing mortality of gastro-esophageal cancer patients [27]. The inhibition of the transforming growth factor-β (TGF-β) signaling pathway mediated by Losartan and its analogs results in a reduced secretion of collagen I and consequently reduced desmoplasia, improving the delivery of chemotherapeutics to tumor cells [28,29]. Similarly, Ronespartat (SST0001), n heparanase inhibitor with a completed Phase 1 clinical trial for the treatment of multiple myeloma (Clinical Trial NCT01764880), showed promising results in inhibiting tumor growth when used alone or in combination with other TME targeting agents [30,31]. However, agents that degrade and/or deconstruct ECM must be used carefully, since they may induce metastasis instead of avoiding tumor progression [32].

The increased expression of matrix metalloproteinases (MMPs) and collagen cross-linkers are also preponderant for the modulation of ECM within the TME and are generally connected to a poor prognosis [24,26,33]. Indeed, MMPs are major players in cell invasion, since they are responsible for proteolysis and detachment of tumor cells from the ECM, resulting in cancer stem cell (CSC) formation and metastasis (reviewed in [26]). Polymorphisms in promoter regions of MMP3 and MMP1, and particularly the MMP1 -1607 1G>2G polymorphism, are risk factors for tumor development and progression [34,35,36,37]. Hence, it is not surprising that several drugs targeting MMPs have been developed. For example, Incyclinide, also known as CMT-3 and COL-3, is an MMP inhibitor that went through several clinical trials for advanced carcinomas (Clinical trials NCT00004147, NCT00003721, NCT00001683, and NCT00020683) [38,39,40]. Other MMPs targeting strategies include JNJ0966, highly selective towards MMP-9, and the antibody Fab 3369 that targets MMP-14 [41,42].

### 2.2. Targeting Hypoxia and Acidosis

The fast proliferation of tumor cells is associated with a high requirement of oxygen, which cannot be sustained by the surrounding blood supply, resulting in limitations of oxygen availability to the cells and hypoxia. The partial oxygen pressure within the TME is generally lower than in normal tissues. In addition, the TME may experience two types of low oxygenation events, chronic and acute/cycling hypoxia [18,43]. Chronic hypoxia arises when the diffusion of oxygen is limited by distended diffusion distances or vein geometries that difficult diffusion, while acute hypoxia emerges when transient perfusions occur, such in vascular occlusion caused by cell aggregates [18].

Hypoxia triggers a series of cellular responses to counteract the oxygen deficit experienced by the cell, mainly coordinated by the transcriptional factor hypoxia-induced factor-1 (HIF-1) [44]. HIF-1 is a dimeric protein complex composed by two subunits that are constitutively expressed in the cells, the cytoplasmic-oxygen dependent subunit alpha, HIF-1α, and the nuclear subunit beta, HIF-1β [44]. In physiological oxygen concentrations, prolyl-hydroxylase enzyme-2 (PHD-2) hydroxylates the oxygen dependent-degradation domain of HIF-1α, with subsequent ubiquitination and degradation of the subunit [19]. Under hypoxia, the lower activity of PHD-2 allows the translocation of HIF-1α to the nucleus, interacting with the HIF-1β subunit and, together with the transcriptional co-activators P300 and CREB binding protein (CBP), binds to the hypoxia-responsive elements located at the promoter regions of over 100 genes involved in hypoxia response [19,44]. This response to low oxygen pressure levels includes regulation of genes involved in glucose metabolism, cell proliferation, angiogenesis, macrophage polarization into tumor-associated macrophages (TAM) and metastasis (reviewed in [11,19]). Several compounds and therapies were designed to tackle HIF-1 or its targets to avoid tumor progression (reviewed in [45,46]). Among these, Topotecan is a topoisomerase 1 inhibitor, FDA approved and used as second line treatment of ovarian and small cell lung cancer. Topotecan has been studied in a clinical trial for treatment of refractory advanced solid neoplasms expressing HIF-1α (NCT00117013) [47]. In addition, other clinical trials include: evaluation of Metformin effect in tissue oxygenation of head and neck squamous cell carcinoma (NCT03510390); the phase 4 trial study of the effect of Everolimus (RAD001) in patients with advanced renal cell carcinoma (NCT01206764); the phase 2 study of the effect of Everolimus (RAD001) conjugated with Lenvatinib in renal cell carcinoma (NCT03324373); and the phase 2 study of pharmacodynamics of Digoxin (DIG-HIF1) in newly diagnosed operable breast cancer (NCT01763931).

To survive in an oxygen and nutrient deficient environment, and to respond to the energy demands of their high proliferation rate, tumor cells adjust their glucose metabolism from the highly efficient oxidative phosphorylation to the less efficient glycolytic pathway, a process named “Warburg effect” [20,21]. According to the Warburg effect, even in the presence of adequate amounts of oxygen, tumor cells obtain energy (in terms of ATP production) predominantly via the anaerobic glycolytic pathway rather than the oxidative phosphorylation [20,21]. This metabolic shift is mediated by the HIF-1 transcriptional regulators that, under hypoxia conditions, induce glucose transporters (GLUTs) to increase glucose import, and regulate the metabolism, favoring anaerobic glycolysis over oxidative phosphorylation [11,19,21]. The preference for anaerobic glycolysis by tumor cells, results in the intracellular accumulation of lactic and carbonic acid [20,21,48]. To prevent the toxic intracellular acidification, tumor cells trigger the expression of proton extrusion mechanisms, including proton flux regulators, such as vacuolar H^+^-ATPases, Na^+^/H^+^ exchanger, monocarboxylate transporters, carbonic anhydrase IX and Na^+^/HCO_3_ co-transporters (reviewed in [48,49,50,51]). The increased acidity in the TME together with low oxygenation and low nutrient supply, result in the selection of tumor cells capable to survive in this hostile environment. Moreover, the pH reduction of the TME confers a survival advantage to tumor cells, as the cytotoxic environment of the TME limit the number and ability of immune cells to inhibit tumor progression [12]. To avoid acidification within the TME, clinical researchers have been focused on the inhibition of proton exchangers and transporters (reviewed in [48]) and of carbonic anhydrase (reviewed in [48,49,52,53,54,55,56]). 

Acidification of the TME seems to protect tumor cells from chemotherapy, since the alteration of the pH partitioning at the cell membrane results in the extracellular accumulation of the chemotherapeutic drugs that ought to enter cells via passive diffusion [50]. To ensure an efficient drug delivery to tumor cells in an acidic TME, current clinical trials propose using combined therapies that target carbonic anhydrase and tumor cells, including the use of Acetazolamide and radiotherapy for small cell lung cancer treatment (NCT03467360), use of SLC-0111 and Gemcitabine for treatment of pancreatic ductal cancer positive for carbonic anhydrase IX (NCT03450018) and combination of Acetazolamide and Temozolomide for treatment of malignant glioma of brain (NCT03011671).

### 2.3. Avoiding Neovascularization—Targeting Endothelial Cells and Pericytes

In the first stages of epithelial tumors, the disordered agglomerate of proliferating tumor cells is separated from the connective tissue by a basal lamina, which limits the oxygen and nutrient supply to the highly proliferative cells. Hypoxia of growing tumor cells will induce the release of angiogenesis signals, such as vascular endothelial growth factor A (VEGFA), that induce angiogenesis response after binding to the VEGF receptor 2 (VEGFR-2) located at the surface of endothelial cells present in neighbor endothelial cells [57,58,59]. The vasculature expansion proceeds by the movement of tip cells containing filopodia for guidance through pro-angiogenic signals and avoiding other cells in a process mediated by the delta ligand like-4 (DLL4) and angiopoietin 2 (ANGPT2) [57,60]. During sprouting, the vessels’ lumen is formed by pinocytosis of several vesicles on the plasma membrane of endothelial cells. The contact with ECM and adjacent cells mediated by integrins in collagen and fibrin matrices will help the coalescence of the vesicles in endothelial cells to form an intercellular lumen [60]. The multiple angiogenic signals triggered by tumor cells result in the formation of heterogeneous blood vessels with chaotic branching structures [11]. Newly formed vessels undergo maturation by consolidation of endothelial cells adhesions, which is achieved by recruitment of the perivascular cells, pericytes and vascular muscle cells. Pericytes are cells of mesenchymal origin that are recruited to the sprouting vessels by platelet-derived growth factor B (PDGF-B) secreted by tip cells and interact with endothelial cells through ANGPT-TIE2 system [57]. When embedded in the basement membrane of the small caliber vessels, pericytes secrete endothelial cells growth factors, MMP inhibitors and ECM molecules, promoting the survival of endothelial cells, while restraining their proliferation [57,60]. Secretion of neural cell adhesion molecule 1 (NCAM 1) and NG2 proteoglycan by attached pericytes induces further pericyte recruitment, contributing to vascular maturation. The increased expression of VEGFA at the tumor location triggers the expression of ANGPT2 by endothelial cells, competing with ANGPT1 for TIE2 on endothelial cells, disrupting the pericyte–endothelial cell interaction, and consequent detachment of pericytes from the basement membrane of tumor blood vessels [57,59]. Hence, high concentrations of VEGFA at the TME induce neo-angiogenesis and promote an immature phenotype of the vasculature, resulting in dysfunctional blood vessels with irregular and leaky lumen [57,58]. This will lead to an increased interstitial fluid pressure and uneven blood flow within the TME [11]. In turn, these events further increase hypoxia and contribute to potentiating tumor development. 

Several antiangiogenic drugs have been tested in clinical trials and showed an increased overall survival or progression-free survival of patients (reviewed in [61]). Bevacizumab (Avastin), an antibody that targets VEGF, was the first anti-angiogenic approved by FDA that is already in the clinics [62]. The fluorescent form of Avastin, Bevacizumab-IRDye800CW, has been showing promising results for its use in tumor imaging [63,64]. Concerning the therapeutic outcome of such drugs, although they showed to improve patient survival, it has not been as great as expected, mostly related to the fact that patients stop responding or do not respond at all to these therapies, or even due to the side effects, for instance, the increased risk of arterial thromboembolic events, which is currently under clinical trials (NCT03709771) [61,65]. However, the efficacy and potentiality of angiogenesis inhibition for tumor treatment is highlighted when a search in the U.S. National library of medicine (http://clinicaltrials.gov, accessed on 18 January 2019) for interventional clinical trials that are recruiting or not yet recruiting, using VEGF and cancer as keywords, retrieved 205 studies. Several of these studies are phase 2 studies for second line treatment of advanced tumors designed with an anti-angiogenic agent, targeting VEGFR or VEGF, and with chemotherapy directed towards tumor cells (e.g., NCT01744249, NCT02704767 or NCT03503604). However, some studies have tested novel combination strategies for pre-operative therapy (e.g., NCT03395899). Interestingly, most studies are focused on the application of Avastin, a tyrosine kinase inhibitor of VEGFR2, and Bevacizumab (Apatinib) as adjuvants in a cocktail chemotherapeutic treatment of advanced or recurrent tumors (Table 1). Moreover, while some studies evaluate the action of two anti-angiogenic agents, e.g., NCT01684397 that evaluates the effect of Pazopanib and Bevacizumab (Avastin) in treating patients with metastatic kidney cancer, other studies compare their efficacy. One such example is the phase 2 trial that compares the action of Bevacizumab (Avastin), targeting VEGF, versus the action of Apatinib, targeting VEGFR2, in colorectal, intestinal, gastrointestinal and digestive system neoplasms (NCT03271255). Another example is the phase 3 trial NCT03533127 comparing the action of two anti-VEGF antibodies, the novel LY1008 and the FDA approved Bevacizumab (Avastin), when combined with Paclitaxel and Carboplatin for non-small cell lung cancer (NSCLC) therapy. Table 1 lits the therapeutic agents that target angiogenesis and are currently in clinical phase 3 or 4 trials, alone or combined with chemotherapeutic agents targeting tumor cells.

Besides using drugs to directly target VEGF or VEGFR, multireceptor tyrosine kinase inhibitors are also used for VEGFR, PDGFA and -B receptors and c-Kit inhibition. One example is Pazopanib (Votrient), which is FDA approved and commonly used in clinical practice for treatment of advanced renal cell carcinoma and soft tissue sarcoma [66,67,68]. Other examples include Sunitinib, with a c-KIT– inhibitory activity, and sorafenib, an antiangiogenic tyrosine kinase inhibitor that also targets Raf kinase activity [61]. Everolimus (RAD001), a rapamycin derivative m-TOR inhibitor, is being tested in a phase 4 clinical trial to inhibit tumor growth and reduce angiogenesis in renal cell carcinoma patients (NCT01206764, Table 1). The inhibition of m-TOR will indirectly decrease endothelial cells proliferation, and hence angiogenesis, through the mTOR/AP-1/VEGF pathway [69].

### 2.4. Targeting Immune System

The TME shows a great diversity in different types of cancers, in regards to the composition of immune cells. While some tumors present few signals of inflammation, others show a high number of immune cells either at the periphery or infiltrated within the tumor [70,71]. Cells from both arms of the immune system can be found within the TME and, depending on the molecular signals within the TME, the same immune cell type may inhibit or promote tumor progression [12,23].

The environment surrounding tumor cells is characterized by the chronic overexpression of inflammatory mediators, and the immune system struggles to recognize aberrant cells and remove them, i.e., immune cells become unresponsive to tumor cells [17]. Considering the role of immune system in cancer, several routes could be used to tackle tumor progression: (1) inhibition of macrophage recruitment into tumor tissues; (2) inhibition of macrophage differentiation into the pro-tumoral phenotype (TAMs); (3) target chronic inflammation or pro-tumorigenic factors supplied by adaptive immune cells; and (4) activate the anti-tumoral activity to circumvent the risk of developing cancer or a poor prognosis of the patient when a tumor is already established [72]. 

#### 2.4.1. Inhibiting Macrophages Recruitment and Differentiation

Monocytes, originated in bone marrow and spleen, are recruited to tumors by both malignant stromal or tumor cell-derived chemokines and growth factors, and can differentiate in two subsets of macrophages, the M1-type macrophages that are activated by interferon gamma (IFN-γ), and the M2-type macrophages that are induced by exposure to cytokines, such as interleukin 4 (IL-4), IL-10, TGF-β, Ggranulocyte-macrophage colony stimulating factor (GM-CSF), Aannexin A1, or tumor cell-surface molecules [73,74,75]. M1 macrophages express high amounts of pro-inflammatory stimuli such as inducible nitric oxide synthase (iNOS) and tumor necrosis factor alpha (TNF-α), and act as antigen presenting cells for antibody production against foreign proteins [73,74]. On the other side, TAMs generally present the M2 subtype [73]. The polarization of macrophages is crucial for cancer prognosis. By secreting cytokines such as IL-6, IL-18, TNF-α, TNF-β, IL-1β, IFN-γ and IL-23, arginase 1 (ARG1) and ECM-modifying enzymes into the TME, TAMs contribute to tumor cell growth, angiogenesis, invasion and metastasis [73,76]. The cellular stress experienced by tumor cells, including oxidative, proteotoxic, mitotic and metabolic stress, seems to be preponderant on the polarization of macrophages [12,74,77]. A high number of TAMs at the TME is generally accompanied by a poor prognosis [12,74,78].

The myeloid-derived suppressive cells (MDSCs) are important players in the immune response against tumors [23,79]. The elevated release of growth factors such GM-CSF and VEGF by cancer cells induce the production of MDSCs in the bone marrow that are kept undifferentiated in the TME [80]. The presence of MDSCs at the TME is usually associated with poor prognosis [81]. MDSCs are involved in tumor progression by promoting tumor proliferation, inducing vascularization, and by secretion of ARG1 and iNOS that suppress the anti-tumorigenic dendritic cells, T cell activation and natural killer (NK) cell cytotoxicity [23,79,81]. MDSCs are precursors of dendritic cells, macrophages and granulocytes, and they can differentiate into TAMs under hypoxic conditions [81,82].

Szebeni et al reviewed in detail the emerging molecule-based therapies focused on TAMs and MDSCs [83]. Overall, macrophage-targeting strategies show therapeutic efficacy; nonetheless, when used in combination with other therapeutic approaches, such as conventional cytoreductive therapies, angiogenesis inhibitors, and immunotherapy, better results are achieved. Additionally, other strategies involving the targeting of macrophages-recruiting mediators have been explored, which includes chemokines, complement components, colony-stimulating factor-1 (CSF-1) and VEGF [84,85]. Similarly, the inhibition of CSF-1 receptor (CSF-1R) is being use as strategy to inhibit tumor proliferation mediated by MDSCs [83,86,87]. The inhibition of CSF-1 signaling using anti-CSF-1R neutralizing antibodies or small molecule inhibitors has been used to decrease infiltration of TAMs and MDSCs and consequently inhibit tumor progression and metastasis [83,85]. The antibody anti-CD204 and a targeted-folate-receptor beta (FRβ) immunotoxin are also able to eliminate TAMs, since CD204 and FRβ are highly expressed on their surface [88].

Table 2 summarizes the therapeutic agents targeting macrophages or MDSCs recruitment that are in interventional clinical trials currently recruiting or not yet recruiting (based on http://clinicaltrials.gov). These novel therapeutic strategies are mainly focused on inhibitors of CSF-1 or CSF-1R alone or combined with other therapeutic agents targeting tumor cells. As for VEGFR targeting, the use of tyrosine kinase inhibitors, such as Nilotinib, is also proposed with the objective of neutralizing CSF-1R and hence reduce the presence of TAMs and MDSCs in the TME.

Chemotherapeutical agents targeting tumor cells can also have effect on TAMs and MDSCs recruitment, differentiation and viability. One example is Vemurafenib, a BRAF kinase inhibitor, FDA approved when combined with the MEK inhibitor Cobimetinib for treatment of metastatic melanoma harboring BRAF V600 mutation, also blocks the recruitment of MDSCs [83,89,90].

#### 2.4.2. Targeting Chronic Inflammation

TAMs secrete cytokines and other inflammatory stimuli responsible for chronic inflammation within the TME [12,91]. For this reason, tumors are frequently regarded as “wounds that never heal” [7,92]. Inflammation may also promote the transformation of early neoplasia to fully developed cancer [93,94,95,96,97]. Chronic inflammation and tumorigenesis are closely related. While the tumor progression often results in chronic inflammation, non-resolving inflammation on a tissue may drive carcinogenesis [98]. The IL-1 cytokine seems to be preponderant in this connection (reviewed in [98]). Hence, to avoid IL-1 induced inflammation, IL-1 receptor (IL-1R) targeting therapies were developed. Table 3 lists the clinical trials focused on IL-1R antagonists that are recruiting or not yet recruiting. Anakinra (Kineret), an FDA approved IL-1 receptor antagonist used in second line treatment of rheumatoid arthritis, showed promising results for the treatment of breast cancer bone metastasis [99,100]. Similarly, Canakinumab, an anti-IL-1β monoclonal antibody commonly used for treatment of inflammatory diseases, showed reduced incidence of lung cancer in a clinical trial (NCT01327846) of the role of IL-1β inhibition in patients with atherosclerosis that had myocardial infarction and with high levels of high-sensitivity C-reactive protein (hsCRP) [101]. Since then, several therapeutic strategies using Canakinumab were proposed for treatment of the highly aggressive tumors NSCLC and Triple negative breast cancer (Table 3).

Phosphomanno-pentose sulfate (PI-88) is a drug that went through clinical trials and targets inflammatory cells (also endothelial cells) [102]. This drug can inhibit heparanase activity, the expression of which is induced by macrophages infiltrating pre-neoplastic/neoplastic lesions [102]. A meta-analysis to find the most suitable adjuvant therapy of hepatocellular carcinoma patients after hepatic resection, transplantation or locoregional ablation therapy, showed that treatment with PI-88 did not improved overall survival [103] and no clinical trials are currently under way.

Several clinical trials focus on the effect of exercise and healthy practices on inflammation (e.g., NCT03429907 and NCT03242902). Moreover, because tumor removal surgery might induce chronical infection resulting in post-operative complications and tumor recurrence, a phase 4 clinical trial not yet recruiting (NCT02746432) proposes the use of insulin therapy during colon cancer surgery.

#### 2.4.3. Activating Anti-Tumoral Activity of Immune System

Lymphoid lineage cells are frequently identified within TME, with a higher incidence in the tumor margin and in draining lymphoid organs [104]. The role of the adaptive immune system in cancer progression is contradictory [23,105]. Infiltrated B cells and regulatory T cells (Treg) in TME establish an immunosuppressive environment by the secretion of cytokines such as IL-10 or TGF-β, and hence promote tumor progression [23,81]. On the other side, the presence on the TME of innate cytotoxic lymphocytes, NK cells and natural killer T (NKT) cells are linked to a good prognosis in many cancers [23]. The immune surveillance of early stage solid tumor is mainly accomplished with NK cells and CD8+ T cells, which induce perforin- and granzyme-mediated apoptosis of the tumor cells, and consequently act in prevention of tumor development [23]. The presence of NK is generally accompanied by a good prognosis of a cancer patient [81]. NK cells secrete anti-tumoral cytokines IL-2 and IL-12 to the TME, as well as IFN-γ that induces activation of dendritic cells [106]. The CD8+ T cells are activated by the antigen presentation mediated by dendritic cells and act on the antigen-bound major histocompatibility class I molecules present on the tumor cell surface [79].

Stimulation of the anti-tumoral activity of the immune system has been widely exploited to prevent tumor progression. Cytokines are used in combination therapies to inhibit response to anticancer monoclonal antibodies targeting TME (the clinical applications of cytokines for cancer immunotherapy is extensively reviewed in [107]). Among the immunotherapeutic strategies, the application of GM-CSF stands-out. This cytokine is secreted by activated T-lymphocytes, fibroblasts, endothelial cells, macrophages and erythroid lineages and stimulates the antigen presentation on macrophages and dendritic cells. By doing so, GM-CSF stimulates the antibody dependent cellular cytotoxicity of anticancer antibodies [107]. Moreover, the use of GM-CSF vaccines would induce the cytokine production by tumor cells and hence enhance the response of the immune system to the unwanted cells. Interestingly, an analysis of the blood of cancer patients treated with GVAX, a GM-CSF vaccine, showed that the vaccine induced thyoglobulin antibodies with associated prolonged survival of cancer patients [108]. While the clinical trials testing the effect of GM-CSF monotherapy or as adjuvant showed positive effects in the induction of immunological activity in NSLC, melanoma or prostate cancer patients, it was not possible to link these effects in a randomized set of patients, as the outcome seems to depend on factors such as cancer stage [107,109,110,111]. Despite the inconsistent effects of GM-CSF, this cytokine was proposed as monotherapy or as adjuvant in 134 interventional recruiting or not yet recruiting clinical trials (based on http://clinicaltrials.gov). A phase 3 clinical trial to treat Ewing sarcoma patients studies the effect of Vigil (Gradalis, Carrollton, TX, USA) on the progression free survival of patients treated with Irinotecan (Camptosar) and Temozolomide (NCT03495921). Vigil composition consists of patient autologous tumor cells extracorporeally transfected with the gene coding for GM-CSF and a short hairpin RNA targeting furin. Another phase 3 clinical trial analyzes the effect of Sipuleucel-T, an autologous cell product that consists of a recombinant fusion protein of prostatic acid phosphatase, PA2024 linked to GM-CSF, in patients with prostate adenocarcinoma (NCT03686683).

Another strategy to enhance the immune response against tumor cells resides in the targeting of immune checkpoint inhibitors. Despite the high number of tumor-associated antigens present in tumor cells due to the genetic and epigenetic alterations intrinsic to the tumorigenic state, the dysregulated expression of immune checkpoint proteins in tumors results in the inhibition of immune system [112,113]. The targeting/inhibition of immune checkpoints is mainly focused on the cytotoxic T-lymphocyte-associated protein 4 (CTLA-4) as well as the programmed death 1 receptor (PD-1) and its ligand PD-L1. The expectation on the use of immune checkpoint target agents for cancer therapy is highlighted by the 754 clinical trials targeting the PD-1/PD-L1 pathway and the 175 clinical trials that focus on CTLA-4 protein (http://clinicaltrials.gov). However, fatal adverse effects for the use of Nivolumab (Opdivo), an anti-PD-1 antibody, are recently reported [114]. The therapeutic potential of agents targeting these immune checkpoints including their phase 3/4 clinical trials are extensively reviewed elsewhere [112,113,115,116,117].

### 2.5. Targeting Cancer-Associated Fibroblasts

The normal function of tissue in an organ requires the migration, proliferation and secretion activity of stromal cells within the ECM [25]. Mesenchymal stromal cells are a highly heterogeneous population of progenitor cells with multiple origins that are present in most tissues of a human adult and have a ubiquitous role on tumor progression [25,118]. Mesenchymal stromal cells possess ECM remodeling properties, including the ability to participate in collagen turnover and to differentiate into nonhematopoietic cells. In addition, they are involved in the tissue repair and regeneration, and in the immune response modulation [25,118]. The wound resemblance of TME recruits mesenchymal stromal cells that try to restore the damaged tissue [25]. As for macrophages, the mesenchymal stromal cells’ response to environmental signals may result in a pro- or anti-inflammatory phenotype that promotes or inhibits tumor proliferation, respectively [25,118,119]. As an example, the presence of TNF, IL-1, IFN-γ and hypoxic conditions in the TME induces the secretion of proangiogenic and immune suppressive factors (including EGF, PDGF, fibroblast growth factor 2, FGF-2, VEGF, IL-6 and IL-10) allowing tumor proliferation [118].

The inflammatory microenvironment of the tumor recruits platelets and other blood-derived hematopoietic cells that secrete a variety of growth factors (e.g., TGF-β1), inducing the differentiation of resident fibroblasts into myofibroblasts, which in a tumor context are called cancer-associated fibroblasts (CAFs), and a response of mesenchymal stromal cells, endothelial and epithelial cells (the later via Epithelial-to-Mesenchymal transition (EMT)) and pericytes [25,120]. CAFs constitute the most abundant stromal cell population in the TME [25]. They secrete growth factors, hepatocyte growth factor (HGF), FGF and chemokines (CXCL12) that not only stimulate growth and survival of tumor cells, but also induce migration of other cells into the TME [7,25,121]. The phenotypic alterations at the level of the alpha smooth muscle actin of CAFs result in increased desmoplasia at the TME ECM [14]. The consequences of CAFs induced desmoplasia are generally related with tumor progression and metastasis in breast, oral, ovarian and pancreatic cancers [25,122,123,124].

CAFs, as the most abundant cell type in TME, have been also explored as a promising target for cancer therapy [61]. One of the contributors to this type of fibroblast phenotype could be the membrane-bound serine protease, called fibroblast activation protein α (FAP). This protease was found to be expressed in the tumor stroma but not in healthy tissues, which makes it an attractive candidate for targeting CAFs [61]. However, phase 1 and 2 clinical trials of Sibrotuzumab, an antibody targeting FAP, did not accomplish a good outcome. The block of the enzymatic activity of FAP with small molecule inhibitors also resulted in lower survival rates for the patients. Currently, three studies involving targeting of FAP are under way (based on http://clinicaltrials.gov, accessed on 16 January 2019), two of them using RO6874281, an interleukin-2 variant targeting FAP. A phase 2 study in patients with advanced solid tumors with or without metastasis is evaluating the therapeutic ability of a combined therapy with Atezolizumab, an anti-PD-L1 antibody, Gemcitabine, Vinorelbine and RO6874281 (NCT03386721). A phase 1 study is evaluating the safety, pharmacokinetics and therapeutic activity of RO6874281 as monotherapy, RO6874281 combined with Trastuzumab, or RO6874281 combined with Cetuximab, for patients with breast and head and neck cancers (NCT02627274). Another phase 1 study is analyzing the effect of re-directed FAP-specific T-cells in FAP-positive malignant pleural mesothelioma (NCT01722149). Novel drugs are continuously emerging to tackle the CAF activity and proliferation, e.g., Conophylline with effect in refractory pancreatic cancers [124]. Even though targeting CAFs seems to be a good strategy, more studies are required to test this hypothesis [61]. 

### 2.6. Targeting Exosomes

As emphasized throughout the last sections, the communication between cells within the TME, be it paracrine (between cells of different types located in the same anatomical region), autocrine (between cells of the same type) or even endocrine (between cells of different types located in different anatomical regions), is critical to modulate the cellular/molecular events involved in maturation of the TME. Resident cells within the TME communicate with each other mainly based on cytokines, chemokines and growth factors. Frequently, the expression of cytokines by tumor cells is accompanied by expression of the respective receptors [105]. However, the communications within the TME are also mediated by vesicles secreted by cells, circumventing the need for cytokines specific receptors.

Exosomes are 30–100 nm vesicles synthesized in the endosomal pathway of both normal and tumor cells. They are composed by a lipid bilayer and are known to carry functional membrane and cytosolic proteins, miRNAs and mRNAs, cytokines, chemokines, growth factors and other signaling molecules [125]. The release of exosomes from normal and diseased cells is affected by multiple factors, including calcium, ischemia, cellular stresses, pH, phorbol esters, and loss of cellular attachment [126,127,128,129,130,131,132]. The content and composition of exosomes varies according to the cell of origin [57]. Once released to the extracellular space, exosomes enable the transfer of proteins, lipids and RNA between different cells, and modulate the recipient cells’ phenotype [133,134]. As exosomes can be found in body fluids, attention has been made to use them as biomarkers of cancer. 

The importance of exosomes in modulation of TME is reflected by the publication of many recent reviews that paid attention to exosomes impact on tumor progression (e.g., [135,136,137,138,139,140]). One of the major features of exosomes is the ability to modulate recipient cells’ phenotype, with deleterious effects in tumor adjacent healthy cells that suffer malignant transition (MT) after internalization of tumor cells derived exosomes (TCDEs) increasing tumoral heterogeneity [133,134,141]. Moreover, TCDEs secretion increases with cancer progression, with epithelial tumor cells secreting less exosomes than tumor cells presenting a mesenchymal phenotype [133,142]. The transport of miRNAs and cytokines mediated by exosomes is known to induce angiogenesis, promote CAF transformation, inflammation and to inhibit lymphocytes or natural killer proliferative response [135]. A study from Zhou and coworkers suggested that the transport of miRNA-21 by hepatocellular carcinoma exosomes contribute for the activation of CAFs and angiogenesis [143]. Moreover, exosomes secreted by early- and late-stage colorectal cancer were both able to induce fibroblasts transformation into CAFs [144]. Interestingly, a proteomic analysis showed that while CAFs activated by early-stage TCDEs showed an upregulation of pro-angiogenic (IL-8, RAB10 and NDRG1) and pro-proliferative (SA1008 and FFPS) proteins, late-stage TCDEs present higher expression of matrix-remodeling proteins (MMP11, EMMPRIN and ADAM10) and regulators of membrane protrusion involved in ECM invasion [144]. In a recent proteomic study, Capello and coworkers suggested that exosomes derived from pancreatic adenocarcinoma present several tumor antigens, functioning as decoys against complement-mediated cytotoxicity [145]. Furthermore, TCDEs play a crucial role in metastasis formation, by inducing EMT of the malignant epithelial cells, promoting dissemination of CSCs throughout the vascular and lymphatic system via chemotaxis, and establishing favorable environments at the potential metastatic location and aid the survival of neoplastic cells at sites of metastasis preparing the niche in the new anatomical location (reviewed in [138,146].

Internalization of TCDEs not only modulate epithelial healthy cells but also all cells present at the tumor neighborhood, such as stromal cells, immune system cells or endothelial cells, which, in turn, secrete harmful exosomes that will also participate in tumor progression [147]. Hence, as the TME matures, the content of malicious exosomes increases exponentially promoting tumor progression. Exosomes are also important mediators of multidrug resistance (reviewed in [148]). Hence, it is not surprising that several strategies focused on the inhibition of malicious exosomes’ biogenesis have been tried; however, this could lead to the inhibition of some other crucial pathways of healthy cells (reviewed in [149]). Silencing of RAB27a, a GTP-binding protein responsible for vesicle trafficking, was proven helpful in reducing the exosome secretion of cancer cells, without consequences on cell viability [133,150,151]. The interventional clinical trials regarding exosomes that are recruiting or not yet recruiting are mainly interested on exosomes as biomarkers for cancer diagnosis (http://clinicaltrials.gov, accessed on 16 January 2019), such as study NCT03608631 that investigates the possible use of exosomes presenting the HSP70 protein at their membrane for early diagnosis of patients with malignant tumors. Another clinical trial focused on cancer biomarkers (NCT03235687) is recruiting prostate cancer patients to evaluate the performance of the ExoDx Prostate (IntelliScore) device, a non-digital liquid biopsy examination based on urine to predict whether the patient requires an invasive biopsy for diagnosis. The NCT02892734 phase 2 trial assesses the ctDNA in blood circulating exosomes to evaluate the potential use of Nivolumab and Ipilimumab for treatment in recurrent stage IV HER2 negative inflammatory breast cancer. Exosomes are also being studied in clinical trials to evaluate their efficacy as vehicles of small interference RNA (siRNA), e.g., the NCT03608631 phase 1 trial that aims to analyze the maximum tolerated dose, dose limiting toxicities, and possible side effects in administrating MSC-derived exosomes with siRNA against KrasG12D in patients with metastatic pancreatic ductal adenocarcinoma with KrasG12D mutation. Another clinical trial has been evaluating the possible use of ginger and *Aloe* derived exosomes to reduce chronic inflammation and insulin resistance in patients with polycystic ovary syndrome (NCT03493984).

## 3. The Case of Combined Therapies

Although the mono-therapy approach is a very common strategy, combined approaches have been extensively explored in clinical trials and they are considered the key for cancer treatment [16]. In the past, these combinations were based on cytotoxic drugs, but, nowadays, this is also being applied to targeted therapeutics, as monoclonal antibodies and small-molecule kinase inhibitors, which seem to be more effective in combinations, as well [152]. Through this kind of approaches, multiple pathways can be targeted, which can avoid MDR and with low associated toxicity [153,154].

In 1965, the first possibility of a combined treatment for pediatric patients with acute lymphocytic leukemia was proposed by Emil Frei and Emil J. Freireich, where a regimen known as POMP regimen (combination of methotrexate, 6-mercaptopurine, vincristine and prednisone) was used and proved to be very successful [155]. After this first approach, many strategies were proposed to simultaneously target different pathways, aiming to create synergistic or additive effects. As the knowledge about the TME and the crosstalk between stromal and tumor cells progressed, different combinatory approaches were defined to target the different cells within the TME at the same time based on the assumption that modulating the tumor environment could more effectively tackle the progression of cancer. For instance, Mangiameli and colleagues applied combined approaches in Ocular Melanoma [156]. This malignancy does not respond to systemic therapy since tumor cells are able to circumvent cytotoxic therapies, whose target is their death. Instead, by targeting the TME, an additive to synergistic effect was achieved, inhibiting the growth of tumors and the development of metastases. The authors used agents to target simultaneously tumor cells and endothelial ones—in this case, Sorafenib (Nexavar™, Bayer) combined with Lenalidomide (Revlimid® Celgene). Sorafenib inhibit multiple receptor tyrosine kinases and Ser/Thr kinases, which includes all isoforms of Raf, all isoforms of VEGFR, and PDGFR-β, present in tumor cells and also in its surrounding vasculature and Revlimid is an immune modulatory drug that targets the immune system and other pathways including caspase-mediated apoptosis of cancer cells and prevents neovascularization [156]. The results suggest that combining these two drugs was a viable strategy leading to the inhibition of growth of ocular melanoma xenografts in mice, including in highly aggressive models where metastases were already developed [156]. More recently, Kitano and colleagues established a combination therapy regarding renal cell carcinoma [157]. In this study, Sunitinib was used to inhibit tyrosine kinase activity of VEGF and PDGF receptors, which are overexpressed by stromal cells, and Everolimus inhibits the mTOR pathway, involved in cellular processes such as cell growth and proliferation, cell metabolism, and angiogenesis. Although Sunitinib alone only decreased stromal reactivity and Everolimus only decreased tumor growth, when combined they reduced both the growth rate and stromal reaction [157]. Overall, these results revealed that such combinations were promising approaches for the modulation of the TME, inhibiting both tumor and stromal cells. Nowadays, clinical trials evaluating an agent targeting only one TME component are rare, as depicted in Table 1, Table 2 and Table 3. 

To increase the efficacy of anti-angiogenic therapy based on VEGF and VEGFR2 inhibition, Allen and coworkers treated refractory pancreatic, breast and brain tumor mouse models with combined therapy using PD-1/PD-L1 pathway blockers and anti-angiogenic agents, since an increased expression of PD-L1 was observed after anti-angiogenic treatment [158]. Interestingly, they found that anti-PD-1 therapy sensitized and prolonged the efficacy of the anti-angiogenic therapy in pancreatic and breast cancer models [158]. On the other side, the anti-angiogenic therapy improved anti-PD-L1 treatment, especially by the increased cytotoxic T cell infiltration due to the formation of intra-tumoral high endothelial venules induced by the therapy [158]. Based on the synergistic effects observed by Allen and coworkers, several clinical trials currently recruiting or not yet recruiting analyze the effect of the combinatorial, including phase 3 trials for treatment of NSCLC (NCT03802240), pleural mesothelioma malignant (NCT03762018) and in combination with pegylated liposomal doxorubicin hydrochloride for treatment of ovarian, fallopian tube and peritoneal carcinoma (NCT02839707). Table 4 lists the active clinical trials using this combinatorial approach.

Following Allen and co-worker’s discovery [158], clinical trials have analyzed the use of combined therapies with agents targeting the immune system and angiogenesis. One example is the phase 1 trial NCT02665416 that evaluates the combination of Selicrelumab, a CD40 agonist, with Vanucizumab, a bispecific antibody that targets simultaneously ANGPT2 and VEGF-A versus the action of Selicrelumab with Bevacizumab, an anti-VEGF antibody, for treatment of advanced solid tumors. Other phase 1 and 2 studies assess the advantage of using Natural Killer cells immunotherapy with Bevacizumab for malignant solid tumor treatment (NCT02857920). The phase 2 clinical trial NCT02923739 studies whether the administration of Emactuzumab, an anti-CSF-1R, increases the progression-free survival of Bevacizumab with Paclitaxel combined therapy.

The application of tyrosine kinase inhibitors to tackle simultaneously different TME components is being widely studied for treatment of renal cell carcinoma, breast cancer, ovarian cancer, metastatic colorectal cancer, gastrointestinal cancer, thyroid cancer and NSCLC (reviewed in [159,160,161,162,163,164,165]). Despite the clinical potential of the tyrosine kinase inhibitors for cancer treatment, some side effects, including vascular and gastrointestinal toxicities, are observed, and a significant survival and tumor relapse must be achieved to be clinically advantageous [166,167]. Table 5 lists active clinical trials using tyrosine kinase inhibitors for cancer therapeutics (based on http://clinicaltrials.gov, accessed on 16 January 2019). Most often, studies use the anti-PD1/PDL-1 pathway and anti-angiogenic combined therapy. One example is the application of Lenvatinib that inhibits VEGFR1, -2 and -3, PDGFR, c-Kit and RET proto-oncogene, combined with Pembrolizumab (anti-PD-1) for treatment of endometrial neoplasms (NCT03517449). In the same line, the phase 2 and 3 clinical trial NCT03794440 intends to examine the safety and tolerability of Sintilimab, a PD-1 inhibitor, in combination with the anti-VEGF monoclonal antibody IBI305, versus the combination of Sintilimab with Sorafenib (Nexavar), a tyrosine kinase inhibitor.

The improvement in the efficacy of cancer therapeutics will probably be based on the use of combined therapies targeting all cell populations that cohabit in the TME. Novel tactics can be foreseen in which anti-tumoral and anti-stromal agents are administered together to modulate the surroundings of the tumor at the same time shrinking or even eradicating it, avoiding tumor recurrence and resistance emergence, as well as lowering the toxicity [72]. With this purpose, there are some active clinical studies that evaluate the combination of several therapeutic agents targeting TME components with or without tumor cells targeting, including NCT03193190, an immunotherapy-based treatment combination for treatment of metastatic pancreatic ductal adenocarcinoma patients, or NCT02693535 that analyzes the application of FDA approved targeted therapies for treatment of advanced solid tumors. 

Although targeted therapies and combinations can hold the promise of cancer therapy, where target non-specificity is no longer a major problem (as for conventional therapeutics), the dose achievable within the solid tumor is limited, which also results in toxicity [168,169]. Specifically, for combined therapy, dissimilar pharmacokinetics and biodistribution of drugs is also a problem that should be considered [153].

## 4. Nanomedicines

**Figure 3:**
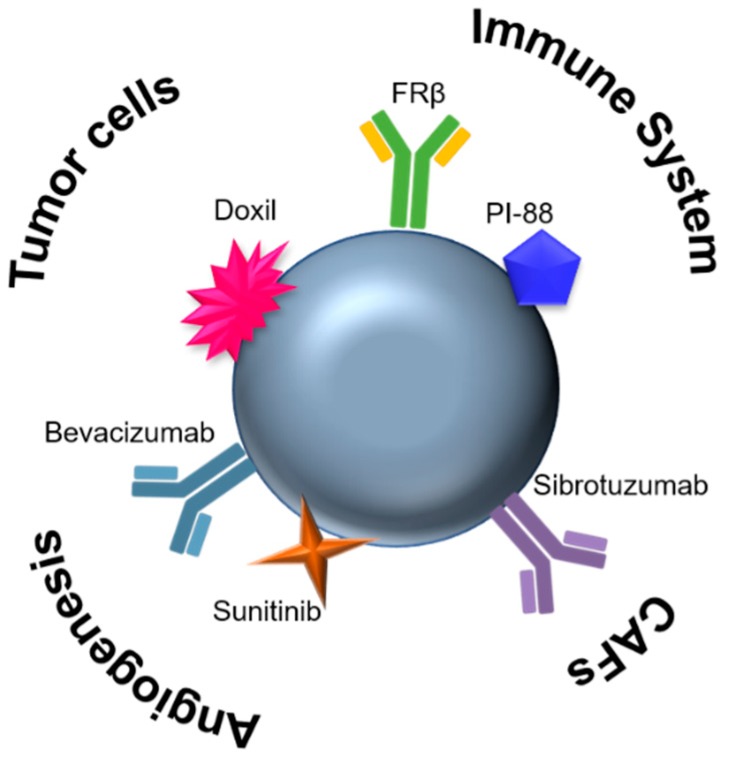
A single nanomaterial (e.g., nanoparticles) can be functionalized with different moieties to target different cell populations in the TME (tumor and stromal cells), enabling a combined strategy for cancer therapeutics. CAFs, Cancer-associated fibroblasts; FRβ, folate-receptor beta; PI-88, Phosphomanno-pentose sulfate.

Nanotechnology has been revolutionizing different scientific fields and medicine is undoubtedly one of them, further coined as nanomedicine [170]. Nanomedicines consist of particles with different sizes and shapes and made of different materials that at nanoscale feature amazing physicochemical properties, which are very interesting to be applied in the diagnostics and therapeutics fields. The materials range from inorganic, such as gold, iron oxide, silver, and silica, to organic, including lipid-based, cell-membrane derived, and layer-by-layer assembled [16,171]. Not only are their size and biocompatibility ideal to circumvent the different barriers that drugs should surpass when systemically administered, but also, due to their chemistry of surface and large surface-volume ratio, different biological moieties can be anchored, tailoring each nanomedicine for different purposes (Figure 3) [16,169,170]. In addition, such nanocarriers allow for a controlled and sustained release of drugs (not only through nanoparticle design but also through triggers that only affects these systems, for instance changes in reduction potential or pH), whose specificity will improve due to an increased internalization and intracellular delivery, which overall contributes to lowering systemic toxicity [171,172,173].

According to the biological moiety that is functionalized on the nanoparticle, these nanosystems can be used for diagnostics, therapeutics and theranostics, which combine both applications in the same system [173]. Theranostics allows, for instance, the detection of a certain biomarker that triggers the release of a drug, while at the same time providing the means to monitor the disease in real time, due to unique optical properties (use as imaging agents, fluorophores or MR contrast agents) [170,172].

Nanomedicine and new models for cancer therapeutics go hand in hand to the development of new targeted strategies towards the efficient delivery of mono and combined therapeutics [174]. At the same time, a single multifunctional nanocarrier may target more than one cell type. Indeed, tailoring nanomedicines profits from the advances in the understanding of cancer physiopathology and TME (including vascular abnormalities, oxygenation, perfusion, pH and metabolic states), towards the fabrication of almost infinitive configurations of nanosystems [170]. In addition, the control pharmacokinetics and dynamics of different drugs can be accomplished, likely giving rise to better outcomes regarding the effect of combinations, since the co-delivering of drugs can greatly decrease the probability of developing multidrug resistance [16]. Besides, nanomedicines can also facilitate conventional approaches by guiding surgical removal of tumors, as well as enhancing radiotherapy [16].

Nanomedicines can achieve solid tumor through passive and/or active targeting, which are connected since active targeting process occurs after passive accumulation within the tumors [168]. This accumulation is due to the enhanced permeability and retention (EPR) effect that occurs since tumor vasculature tends to be leaky and the clearance by the lymphatic system tends to be poor [168,170,173,175]. Regarding active targeting, multiple ligands can be used to functionalize the surface of nanocarriers allowing the recognition of specific receptors expressed at the target site [168,170]. In fact, recent researchers have been focused on tackling TME components using nanotechnology. Mardhian and coworkers conjugated human relaxin-2 (RLX) with superparamagnetic iron oxide nanoparticles (SPION) to inhibit pancreatic stellate cells differentiation into CAF-like myofibroblasts, resulting in a significant inhibition of pancreatic ductal adenocarcinoma growth [176]. Gold nanoparticles coated with anti-angiogenic peptides were proven to inhibit the formation of new vessels in an *ex ovo* chick chorioallanoic membrane assay [177].

Although these systems seem answer most of the mentioned drawbacks concerning conventional and targeted therapeutics, nanoparticles already FDA-approved (Doxil/Caelyx, DaunoXome and Abraxane) are not as remarkable [175]. Despite being able to reduce systemic toxicity, these nanocarriers do not improve the overall survival of patients, leaving plenty of room to improve nanomedicines regarding tumor type and stage, and the properties of TME [172,175]. This means that nanomedicines should to take into account the particle type and cancer, since one-size-fits-all does not seem to work (for more information, please read the commentary from Chauhan and Jain [175]). Wang and coworkers recently reviewed the “tumor on a chip” platforms that facilitate clinical translation of nanoparticles-based cancer therapies [178]. Overchuck and Zheng stated that nanomedicines should be combined with pre-treatment strategies to improve their target achievement, thus not only tailoring nanomedicines’ features with TME cells and conditions, but also, depending on this highly variable TME and its components, subtle changes (pre-treatments such as radiation, hyperthermia and photodynamic therapy) can be applied in the tumor microenvironment to promote the accumulation of such nanoformulations (for more information, please check [173]). Envisaging the improvement of drug delivery, smart-delivery nanoformulations take advantage of the increased expression of MMP at the TME (reviewed in [179]).

Undeniably, nanomedicines are still in route to become the most promising effective anti-cancer treatment, with room for improvement. A few nanoparticle-mediated TME therapies are already under clinical trials, with one already approved, which shows that there is plenty of space to keep exploring them. The one already approved (Liposomal mifamurtide, only in Europe) targets specifically the immune system by activating monocytes and macrophages [180]. Beyond that, there are two more ongoing clinical trials using Mifamurtide, an observational study (NCT03737435) to analyze the tumor microenvironment of patients with localized osteosarcoma treated with mifamurtide, and a phase 2 trial that evaluates the efficacy of Mifarmutide as add-on treatment to post-operative chemotherapy in patients with high-risk osteosarcoma. The nanomedicine CRLX101, a polymeric nanoparticle with Camptothecin that targets HIF-1, is under two recruiting clinical trials, combined with Enzalutamide for treatment of prostate cancer (NCT03531827) or with Olaparib for treatment of relapsed/refractory small cell lung cancer (NCT02769962). Besides these studies, phase 1 trials (NCT00356980 and NCT00436410) are using CYT-6091 consisting of gold nanoparticles with TNFα to target the immune system.

## 5. Models for the Study of TME

**Figure 4:**
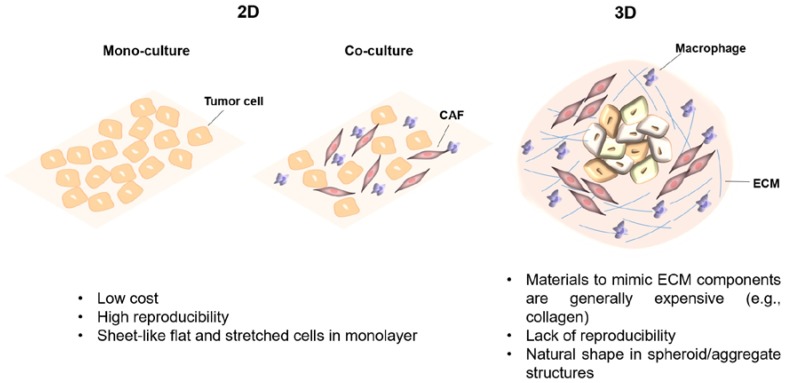
Main properties of in vitro 2D and 3D culture systems [187,188]. CAF, Cancer-associated fibroblast; ECM, Extracellular matrix.

The FDA approved cancer drugs are usually optimized to be highly effective in vitro using cancer cells monolayers and in vivo using mouse xenograft cancer models [181,182,183]. However, considering the complexity of tumors, there is a gap between these two models. In fact, the design of innovative therapies for effective cancer therapy require adequate preclinical models that mimic TME. With that purpose, cell and tissue engineered tumor models have been gaining attention since they can recapitulate more closely the TME to which the cells within the tumor are exposed (e.g., survival, proliferation, gene expression heterogeneity and multidrug resistance), also enabling the control of environmental factors and measurement of cell responses [184,185]. Experiments conventionally start on 2D models, providing initial improvements using monocultures of commercial/immortalized cell lines, in a simple, convenient and relatively reproducible way. These 2D cultures can be improved by using co-cultures of different cell types to better resemble human tissues—cell-to-cell communications [170,186] (Figure 4). To improve even more tissue complexity (mechanical and biochemical signals), mimicking the tumor architecture, 3D (co-)culture systems have been employed (Figure 4) [181,187]. The development of more biologically relevant in vitro tumor models using 3D approaches not only results in improved translation but also contributes to reducing animal testing (three Rs politics) required by the pharmaceutical industry and governmental institutions [187,188].

The reconstitution of the TME in vitro is still a big challenge. The so-called third dimension resembles the architecture of a real solid tumor; nevertheless the functional interactions among tumor, stromal cells and the ECM should also be addressed, since they are key players in tumor progression, invasiveness and metastasis [182]. The currently available 3D culture models of cancer categorized into tumor tissue explants, “tumor on a chip”, and multicellular tumor spheroids (MCTS) are summarized on Table 6.

The tumor tissue explants were the first models to be introduced for in vitro drug discovery and rely on collecting the tumor tissue after biopsy, which is further cleared from necrotic tissue and placed in a collagen matrix [189]. This encapsulation step promotes cell–cell and cell–matrix attachments, proliferation rate, wound tissue expansion, migration and angiogenesis [183]. Afterwards, the tumor is injected with drugs and cultured with media for a desired period [189]. One of the major advantages of using this methodology is the maintenance of the tumor architecture [190]. However, some important disadvantages encompass the difficulty to maintain the culture more than three weeks, as well as the lack of reproducibility concerning tumor heterogeneity [189,190,191]. This last point could be also seen as an advantage since genetic and phenotypic diversity is something very attractive concerning drug screening platforms [192]. In terms of long-lasting culture time, organoid technology can circumvent the limitation featured by tumor explants. In this case, organoid cultures are established from primary tumors, through differentiation of patient-derived pluripotent stem cells or isolated organ progenitors in which specific mutations (to recapitulate the disease-like state on healthy cells) are induced [192]. However, again, there are some drawbacks in using this model since they are made up exclusively of epithelial cancer cells, which poorly resembles both TME and disease progression [192].

Static cell culture systems do not simulate the existing in vivo tumor perfusion, mechanical stimuli and mass transport [192]. Microfluidic systems allow mimicking the cellular vasculature and the movement of biological fluids, improving the similarities to a real TME, where a continuous flow of nutrients and oxygen and removal of waste products exists [189,192,202,203,204]. “Tumor on a chip” is based on the fabrication of a microfluidic device, where simpler to more complex tumor models can be created, from co-cultures of tumor cells with other cell types to organs. This not only allows the study of the role of cell communication in TME, but also the metastatic process as well as pharmacodynamics and kinetics of drugs [189,192]. One example of this technology is a biomimetic liver “tumor on a chip” composed by a decellularized liver matrix and a gelatin methacryloyl in a microfluidics-based 3D dynamic cell culture system [205]. Another example consists in a metastasis-on-a-chip device composed of a microfluidic chamber with cancer cells in a colorectal cancer organoid, connected to multiple downstream chambers with liver, lung and endothelial constructs in an extracellular matrix-derived hydrogel biomaterial [206]. Recent strategies for studying drug sensitivity and toxicity include the use of tumor biopsies to produce tumor organoids in a “tumor on a chip” microfluidic device for personalized therapy [207,208].

Multicellular tumor spheroids (MCTS) or just spheroids are the simplest model of cancer for drug discovery and the most well characterized [189,192]. This model is based on using tumor cells alone or in combination with other cell types [189,201]. Depending on the established interactions between cells, as well as the origin of the cells and cell culture technology, these cellular arrangements would be organized in spherical or other irregular shapes [192]. Besides spatial cell arrangement concerning growth kinetics, ECM reproduction, along with cellular heterogeneity, signaling pathway activity, and gene expression (when MCTS have diameters larger than 500 µM), they also recapitulate very well the physicochemical conditions observed in vivo, such as hypoxia and acidosis [189,192]. Scaffold-based MCTS cell cultures are essentially made up of natural (e.g., collagen), semi-synthetic (e.g., chitosan) or synthetic (e.g., polycaprolactone) hydrogels [184,191], which have the major advantage of reproducing the ECM architecture. Although the use of scaffolds supports the organization of cancer cells, being a source of external signals that promote cell–cell and cell–matrix interactions, these scaffolds do not totally mimic the conditions of the mass transport gradient of a tumor environment [189,191]. However, hydrogels are composed by interconnected microscopic pores that retain the water and hence facilitate the perfusion of oxygen, nutrients, metabolic wastes, growth and other soluble factors [189]. Matrigel is one of the most used commercially available hydrogels, which is derived from secreted basement membrane extracts of Engelbreth–Holm–Swarm (EHS) mouse sarcoma cells and it is rich in ECM components, such as laminin-1 and collagen IV [189,209]. The advantages, disadvantages and respective applications of scaffold-based and free MCTS cell cultures were recently summarized by Nath and Devi [189].

## 6. Conclusions and Outlook

Fundamental research on the understanding of the TME is mandatory for the development of new models that will allow the validation of new therapeutic approaches. The behavior of tumor cells on their own is now known as completely different compared to where they have a microenvironment surrounding them and contributing to a malignant state. This TME has an impact on the expressed surface receptors, activated or silenced signaling pathways and will influence the therapeutic effect/response. To address problems such as no response to therapy or tumor resistance, and aiming at achieving a personalized medicine in oncology, each tumor must be considered as a multifactorial disease, different in each patient and thus requiring a different strategy regarding therapeutics, especially focused on combinations. Finally, the use of nanotechnology can simplify tailored medicine since different targeting molecules can be anchored in a single nanomaterial. 

## Figures and Tables

**Table 1:** Therapeutic agents targeting angiogenesis in interventional phase 3 and 4 clinical trials currently recruiting or not yet recruiting. Data acquired from the U.S. National library of medicine (http://clinicaltrials.gov, accessed on 18 January 2019).

VEGF ^1^/VEGFR ^2^ Targeting Therapeutic Agent	Therapeutic Strategy	Cancer Type	Clinical Trial Reference (Phase)
Bevacizumab (Apatinib)	Bevacizumab (anti-VEGF) in a chemotherapeutic cocktail with 5-Fu, Folinic acid, Panitumumab and intra-arterial vs. intravenous Oxaliplatin	Colorectal neoplasms	NCT02885753 (3)
	Cisplatin with Etoposide vs. Cisplatin, Etoposide and Bevacizumab	Small cell lung cancer	NCT03100955 (3)
	Bevacizumab vs. placebo	Thyroid cancer	NCT03048877 (3)
	Bevacizumab as second line treatment	Intrahepatic Cholangiocarcinoma	NCT03251443 (3)
LY01008 and Bevacizumab	LY01008 (anti-VEGF antibody) with Carboplatin/Paclitaxel vs. Bevacizumab with Carboplatin/Paclitaxel	Non-small cell lung cancer	NCT03533127 (3)
Cediranib	Olaparib (PARP inhibitor) with Cediranib (VEGF-A inhibitor) or Olaparib alone	Ovarian cancer	NCT03278717 (3)
Ramucirumab (LY3009806)	Ramucirumab (anti-VEGFR) with Paclitaxel vs. Placebo with Paclitaxel	Gastric adenocarcinoma	NCT02898077 (3)
Aflibercept	Injection of Aflibercept (anti-VEGF) vs. placebo injection	Ocular melanoma	NCT03172299 (3)
Everolimus (RAD001)	Everolimus (m-TOR inhibitor) alone	Renal cell carcinoma	NCT01206764 (4)

^1^ VEGF, Vascular endothelial growth factor; ^2^ VEGFR, Vascular endothelial growth factor receptor

**Table 2:** Therapeutic agents targeting macrophages and myeloid-derived suppressive cells recruitment in interventional clinical trials currently recruiting or not yet recruiting. Data acquired from the U.S. National library of medicine (http://clinicaltrials.gov, accessed on 16 January 2019).

Therapeutic Agent	Therapeutic Agent Description	Cancer Type	Clinical Trial Reference (Phase)
Pexidartinib (PLX3397)	CSF-1R ^1^ inhibitor	Advanced solid tumors Giant cell tumor Melanoma Pancreatic/Colorectal cancer Gastrointestinal stromal cancer Advanced solid tumors Gastric cancer	NCT02734433 (-) NCT02371369 (3) NCT02975700 (1/2) NCT02777710 (1) NCT03158103 (1) NCT01525602 (1) NCT03694977 (2)
ARRY-382	CSF-1R inhibitor	Advanced solid tumors	NCT02880371 (2)
BLZ945	CSF-1R inhibitor	Advanced solid tumors	NCT02829723 (1/2)
JNJ-40346527	CSF-1R inhibitor	Prostate cancer	NCT03177460 (1)
Emactuzumab	CSF-1R antibody	Squamous cell carcinoma	NCT03708224 (2)
DCC-3014	CSF-1R inhibitor	Advanced malignant neoplasm	NCT03069469 (1)
Chiauranib	Tyrosine kinase inhibitor	Ovarian cancer Small Cell Lung cancer Hepatocellular carcinoma	NCT03166891 (1) NCT03216343 (1) NCT03245190 (1)
IMC-CS4 (LY3022855)	CSF-1R blocking agent	Pancreatic cancer Melanoma	NCT03153410 (1) NCT03101254 (1/2)
Cabiralizumab (FPA008)	CSF-1R antibody	Pancreatic cancer Melanoma/Non-small cell lung cancer/Renal cell carcinoma Resectable biliary tract cancer	NCT03697564 (2) NCT03502330 (1) NCT03768531 (2)
SNDX-6352 (UCB6352)	CSF-1R antibody	Advanced malignant neoplasm	NCT03238027 (1)
PD 0360324	CSF-1 antibody	Ovarian cancer	NCT02948101 (2)
Nilotinib	Tyrosine kinase inhibitor	Malignant solid neoplasms Soft tissue sarcoma	NCT02029001 (2) NCT03784014 (3)
Lacnotuzumab (MCS110)	CSF-1 antibody	Melanoma	NCT03455764 (1/2)

^1^ CSF-1R, Colony-stimulating factor-1 receptor.

**Table 3:** Therapeutic agents targeting interleukin-1 or interleukin-1 receptor in interventional clinical trials currently recruiting or not yet recruiting. Data acquired from the U.S. National library of medicine (http://clinicaltrials.gov, accessed on 16 January 2019).

Therapeutic Agent	Therapeutic Strategy	Cancer Type	Clinical Trial Reference (Phase)
Anakinra (Kineret)	Combined with Nab-paclitaxel, Gemcitabine, Cisplatin	Pancreatic cancer	NCT02550327 (1)
	Alone	Multiple myeloma	NCT03233776 (2)
Canakinumab (Ilaris)	Alone	_1_ NSCLC	NCT03447769 (3)
	Chemotherapeutic cocktail with or without Canakinumab	NSCLC	NCT03631199 (3)
	Possible use of Canakinumab with Spartalizumab and LAG525	^2^ TNBC	NCT03742349 (1)
	Docetaxel with Canakinumab vs. Docetaxel with placebo	NSCLC	NCT03626545 (3)
	Possible use with PDR001	Colorectal cancer/TNBC/NSCLC	NCT02900664 (1)
	Possible use with PDR001, cisplatin, pemetrexed and carboplatin	NSCLC	NCT03064854 (1)
	Possible use with PDR001	Melanoma	NCT03484923 (2)

^1^ NSCLC, Non-small cell lung cancer; ^2^ TNBC, Triple negative breast cancer.

**Table 4:** Therapeutic agents for combinatorial therapy targeting the immune check point Programmed death 1 (PD-1) and angiogenesis in interventional clinical trials currently recruiting or not yet recruiting. Data acquired from the U.S. National library of medicine (http://clinicaltrials.gov, accessed on 16 January 2019).

Therapeutic Strategy	Cancer Type	Phase	Clinical Trial Reference
HLX10 (anti-PD-1 ^1^) + HLX04 (anti-VEGF ^2^)	Solid tumor	1	NCT03757936
SHR-1210 (anti-PD-1) with Bevacizumab (anti-VEGFR)	Gastric and hepatocellular cancer	1/2	NCT02942329
Atezolizumab (anti-PD-L1) with Bevacizumab (anti-VEGF)	Digestive, respiratory and intrathoracic organs tumors	2	NCT03074513
Atezolizumab (PD-L1 inhibitor), Bevacizumab (anti-VEGF) and Cobimetinib (MEK ^3^ inhibitor)	Ovarian and fallopian tube cancer and peritoneal carcinoma	2	NCT03363867
PLD ^4^ with Atezolizumab (PD-L1 inhibitor) vs. PLD with Bevacizumab (anti-VEGF) and Atezolizumab vs. PLD with Bevacizumab	Ovarian, fallopian tube and peritoneal carcinoma	2/3	NCT02839707
Sintilimab (anti-PD-1) with IBI305 (anti-VEGF), Pemetrexed and Cisplatin vs. Sintilimab with IBI305 and Pemetrexed vs. Pemetrexed and Cisplatin	Non-squamous non-small cell lung cancer	3	NCT03802240
Bevacizumab (anti-VEGF) with Carboplatin and Pemetrexed vs. Bevacizumab with Atezolizumab (anti-PD-1), Carboplatin and Pemetrexed	Pleural mesothelioma malignant advanced	3	NCT03762018

^1^ PD-1, Programmed death receptor 1; ^2^ VEGF, Vascular endothelial growth factor; ^3^ MEK, Mitogen-activated protein kinase (involved in cancer cells proliferation); ^4^ PLD, Pegylated liposomal doxorubicin hydrochloride.

**Table 5:** Tyrosine kinase inhibitors used alone or in combinatorial therapy in interventional clinical trials currently recruiting or not yet recruiting. Data acquired from the U.S. National library of medicine (http://clinicaltrials.gov, accessed on 16 January 2019).

Tyrosine Kinase Inhibitor	Inhibited Tyrosine Kinases	Therapeutic Strategy/Objective	Cancer Type	Phase	Clinical Trial Reference
Sitravatinib (MGCD516)	c-Met, AXL, MER, VEGFR ^1^, PDGFR ^2^, DDR2, TRK ^3^, Eph ^4^	Dosage and clinical activity of Sitravatinib	Advanced cancer	1/1b	NCT02219711
		Sitravatinib with Nivolumab (Opdivo, anti-PD-1 ^5^)	Renal cell cancer	1/2	NCT03015740
Axitinib (AG-013736)	VEGFR1-3, c-KIT, PDGFR	Avelumab (anti-PD-1) with Axitinib	Non-small cell lung or urothelial cancer	2	NCT03472560
		Sandostatin LAR with Axitinib vs. with placebo	Neuroendocrine tumors	2/3	NCT01744249
Cabozantinib	c-Met, VEGFR	Nivolumab (anti-PD-1) vs. Nivolumab with Cabozantinib	Renal cell carcinoma	3	NCT03793166
Lenvatinib	VEGFR1-3	Lenvatinib with Pembrolizumab (anti-PD-1) vs. Paclitaxel or Doxorubicin	Endometrial neoplasms	3	NCT03517449

^1^ VEGFR, Vascular endothelial growth factor receptor; ^2^ PDGFR, Platelet-derived growth factor receptor; ^3^ TRK, Tropomyosin receptor kinase; ^4^ Eph, Ephrin receptor; ^5^ PD-1, Programmed death receptor 1.

**Table 6:** Summary of currently available 3D culture models of cancer.

Culture Model	Composition	Major Advantages	Major Disadvantages	References
Tumor tissue explants	Tumor collected from a biopsy and placed on a collagen matrix	Maintenance of tumor architecture	Difficulty on maintaining the culture for more than 3 weeks	Reviewed in [183,189,190,191,192]
	Organoid cultures from tissue explants	Long-lasting culture	Poorly resembles TME ^1^ and disease progression	
“Tumor on a chip”	co-cultures of tumor cells with other cell types to organs	TME ^1^ reproduction with the movement of biological fluids	Size limited	Reviewed in [193,194,195,196,197,198,199,200]
Multicellular Tumor Spheroids (MCTS)	Spheroids composed by mono- or co-culture aggregates	TME ^1^ reproduction	Fail in reproducing ECM architecture	Reviewed in [184,189,191,192,201]
	Spheroids composed by mono- or co-cultures on a scaffold	TME ^1^ reproduction ECM ^2^ architecture reproduction	Low reproducibility Cost	

^1^ TME, tumor microenvironment; ^2^ ECM, extracellular matrix.

## Abbreviations

ANGPT	Angiopoietin
ARG1	Arginase 1
CAFs	Cancer-associated fibroblasts
CSC	Cancer stem cell
CSF	Colony stimulating factor
CTLA-4	Cytotoxic T-lymphocyte-associated protein 4
ECM	Extracellular matrix
EGF	Epidermal growth factor
EMT	Epithelial-to-mesenchymal transition
EPR	Enhanced permeability and retention
FAP	Fibroblast activation protein
FGF	Fibroblast growth factor
FRβ	Targeted-folate-receptor beta
GM-CSF	Granulocyte-macrophage colony stimulating factor
HGF	Hepatocyte growth factor
HIF-1	Hypoxia-induced factor-1
IL	Interleukin
IFN	Interferon
iNOS	Inducible nitric oxide synthase
MCTS	Multicellular tumor spheroids
MDR	Multidrug resistance
MDSCs	Myeloid-derived suppressive cells
MMPs	Matrix metalloproteinases
MT	Malignant transition
NSCLC	Non-small cell lung cancer
NK	Natural killer
NKT	Natural killer T
PD-1	Programmed death 1 receptor
PDMS	Polymethylsiloxane
PlGF	Placental growth factor
PDGF	Platelet-derived growth factor
PHD-2	Prolyl-hydroxylase enzyme-2
TAM	Tumor-associated macrophage
TCDEs	Tumor cells derived exosomes
TME	Tumor microenvironment
TGF	Transforming growth factor
TNF	Tumor necrosis factor
Treg	Regulatory T cells
VEGF	Vascular endothelial growth factor
